# The establishment of kidney cancer organoid line in drug testing

**DOI:** 10.1002/cam4.7432

**Published:** 2024-06-26

**Authors:** Ryan Tsz‐Hei Tse, Christine Yim‐Ping Wong, Xiaofan Ding, Carol Ka‐Lo Cheng, Chit Chow, Ronald Cheong‐Kin Chan, Joshua Hoi‐Yan Ng, Victor Wai‐Lun Tang, Peter Ka‐Fung Chiu, Jeremy Yuen‐Chun Teoh, Nathalie Wong, Ka‐Fai To, Chi‐Fai Ng

**Affiliations:** ^1^ S.H. Ho Urology Centre, Department of Surgery The Chinese University of Hong Kong Hong Kong China; ^2^ Department of Surgery The Chinese University of Hong Kong Hong Kong China; ^3^ Department of Anatomical and Cellular Pathology The Chinese University of Hong Kong Hong Kong China; ^4^ Department of Pathology Pamela Youde Nethersole Eastern Hospital Chai Wan Hong Kong

**Keywords:** 3D culture, disease modeling, drug screening, organoid model, renal cell carcinoma

## Abstract

**Introduction:**

Kidney cancer is a common urological malignancy worldwide with an increasing incidence in recent years. Among all subtypes, renal cell carcinoma (RCC) represents the most predominant malignancy in kidney. Clinicians faced a major challenge to select the most effective and suitable treatment regime for patients from a wide range of modalities, despite improved understanding and diagnosis of RCC.

**Objective:**

Recently, organoid culture gained more interest as the 3D model is shown to be highly patient specific which is hypothetically beneficial to the investigation of precision medicine. Nonetheless, the development and application of organotypic culture in RCC is still immature, therefore, the primary objective of this study was to establish an organoid model for RCC.

**Materials and Methods:**

Patients diagnosed with renal tumor and underwent surgical intervention were recruited. RCC specimen was collected and derived into organoids. Derived organoids were validated by histological examminations, sequencing and xenograft. Drug response of organoids were compared with resistance cell line and patients' clinical outcomes.

**Results:**

Our results demonstrated that organoids could be successfully derived from renal tumor and they exhibited high concordance in terms of immunoexpressional patterns. Sequencing results also depicted concordant mutations of driver genes in both organoids and parental tumor tissues. Critical and novel growth factors were discovered during the establishment of organoid model. Besides, organoids derived from renal tumor exhibited tumorigenic properties in vivo. In addition, organoids recapitulated patient's in vivo drug resistance and served as a platform to predict responsiveness of other therapeutic agents.

**Conclusion:**

Our RCC organoid model recaptiluated histological and genetic features observed in primary tumors. It also served as a potential platform in drug screening for RCC patients, though future studies are necessary before translating the outcomes into clinical practices.

## 1 INTRODUCTION

### 1.1 Epidemiology of kidney cancer

Kidney cancer ranked 16th and 17th in worldwide incidence and mortality rates. It accounts for 2% of global cancer burden. In Asia, there were more than 150,000 new cases and 80,000 deaths and over 430,000 new cases worldwide in 2020.^1^, ^2^, ^3^ Incidence of kidney cancer is apparently not as high as other urological malignancies, such as prostate cancer and urinary bladder cancer, it is regarded as the most lethal urological cancers though.^4^ The most predominant type of kidney cancer is renal cell carcinoma (RCC), which makes up around 85% of all diagnosed cases.^5^ Of note, it is realized that RCC is a malignant condition with multiple entities. Different types of tumors arising from various parts of nephron can possess a distinct genetic characteristics, histological features and clinical phenotypes. Typical subtypes of RCCs include clear cell RCC (ccRCC), papillary RCC (pRCC) and chromophobe RCC (chRCC). While other uncommon histological subtypes include cystic‐solid, collecting ducts (Bellini), medullary, Xp11 translocation, mucinous tubular spindle cell, associated with neuroblastoma and unclassified.^6^

### 1.2 Challenges of renal cell carcinoma management

Oncologists and clinicians faced numerous obstacles in RCC management. Besides being nearly uniformly resistant to available chemotherapy agents, systemic therapies in RCC also faced several challenges.^7^ It is observed that significant intertumoral and intratumoral heterogeneities exist in advance RCC. Therefore, potentially causes a good initial response to first‐line medications, yet emerging more resistant‐relapse clones which are more difficult to target. In addition, patients harbored different mutational profiles of tumors even they are diagnosed of same subtype of RCC. Therefore, patients' responses to systemic therapies are not equal, which some give satisfactory responses while the others give minimal responses. Of note, discrepancies in response rates were also related to racial differences.^8^ As for advance or metastatic RCC (mRCC), the complex immunosuppressive TME, especially in brain metastasis, influences decisively the clinical response to systemic treatments.^9^, ^10^ As a result, the responses of advanced RCC to immunomodulatory drugs remain speculation and underlying immunological and molecular mechanisms require further studies. Therefore, detail mechanisms of novel immunomodulatory agents should be studied in a more patient‐specific and sustainable model.^11^, ^12^

### 1.3 Three‐dimensional organoid culture

In 2009, Sato et al. discovered a single leucine‐rich repeat containing G‐protein‐coupled receptor 5‐positive intestinal stem cells. It possessed the ability to generate a continuously expanding, self‐organizing and physiological epithelial structure that was similar to normal gut tissue and was named an “organoid” culture. Liu et al. and Chapman et al. then demonstrated that the combination of Rho‐kinase (ROCK) inhibitors and feeder fibroblast culture conditions allows infinite growth of multiple primary human epithelial cell types. Based on this, organoids derived from normal and tumor cells might be able to proliferate indefinitely in vitro, without the need for transducing exogenous viral or cellular genes.^13^, ^14^, ^15^ Nowadays, organoids are increasingly being employed as a model in which to study tumor genetics, as 3D organoid models are able to overcome 2D culture limitations, by recreating the microarchitecture and including a more specific range of secretomes achieved by recapitulating cell–cell and cell–ECM interaction in the human body environment.

**FIGURE 1:**
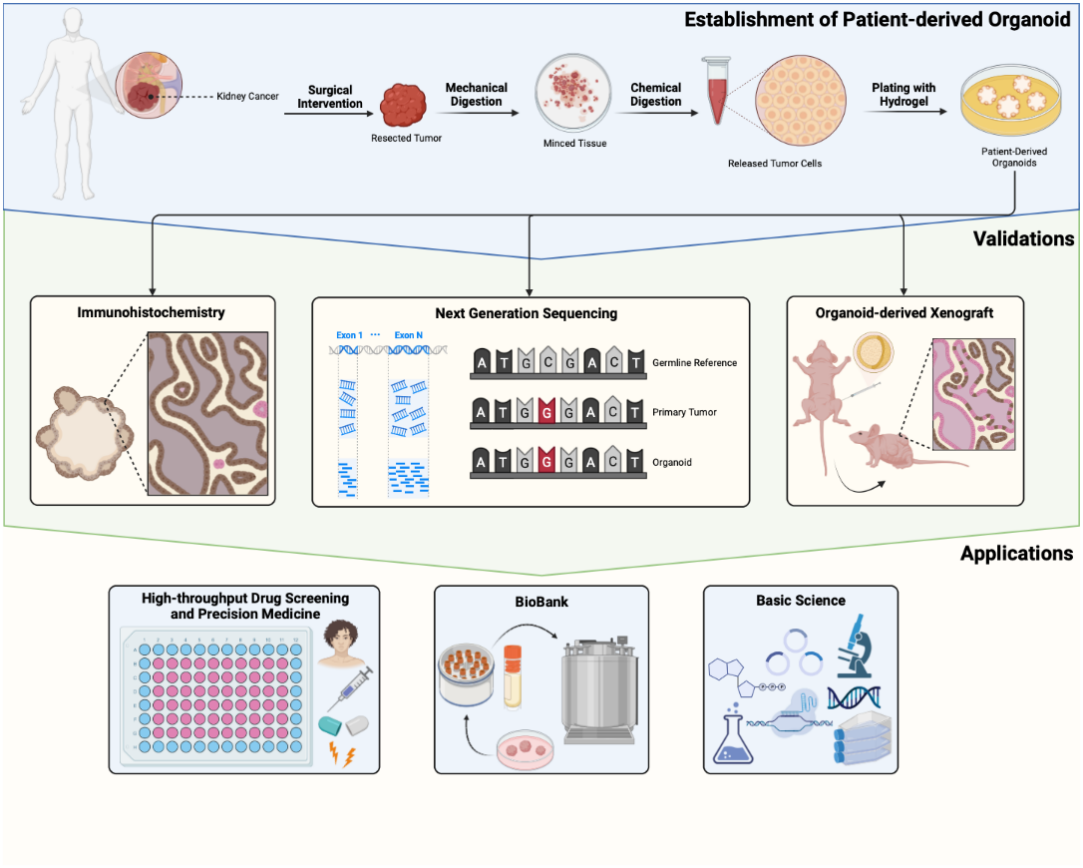
Schematic of establishment of organoid, validation methods, and potential applications. (Created in BioRender.com).

Patient derived organoid models have been applied in cancer research in different urological cancers by different groups and the results had primed us to a new understanding of cancer biology and tumor genetics.^16^, ^17^, ^18^ Nonetheless, limited studies focused on the establishment and application of RCC organoid models. The development of patient derived RCC organoid model was in fact just started to be elucidated since 2015. Although different studies depicted their RCC organoids since then, their results embraced certain obstacles and limitations.^19^, ^20^, ^21^ As a result, in this study, we attempted to derive organoid model from renal tumors and comprehensively validate them in vitro and in vivo alongside the applications of our model in drug screening (Figure 1).

## 2 METHODS

### 2.1 Study design

Patients were recruited in the Prince of Wales Hospital (Hong Kong). All patients were diagnosed with renal tumor and underwent surgical intervention (i.e., partial or radical nephrectomy). RCC specimen was collected from piecemeal of resected tumor while normal tissues were sampled at a distance from the tumor. Tissue samples were used for the establishment of primary organoid cultures and for analysis of parental tumor tissues. Pre‐operative blood was collected from patient for DNA extraction in subsequent WES analysis. A written informed consent was signed by each participant and their legal guardians before any assessments. Clinical Research Ethics Committee of the University and Hospital have granted approval for this study (CREC Approval Number 2014.468).

### 2.2 Tissue dissociation and organoid culture

Tumor tissue were washed with PBS. Tissue dissociation was performed first by mechanical mincing with sterile scissor. Followed by enzymatic digestion in advanced DMEM/F12 (Life Technologies) supplemented with 0.1 mg/mL collagenase from *Clostridium histolyticum* (Type XI) (Sigma‐Aldrich) and 0.125 mg/mL dispase II (Life Technologies) for 1 h at 37°C. Dissociation was terminated with Roswell Park Memorial Institute (RPMI) 1640 Medium (Life Technologies) supplemented with 10% Fetal Bovine Serum (FBS, Life Technologies).

Dissociated tissue mixture was triturated by vigorous pipetting, followed by passing through a 70 μm cell strainer (Corning). Filtered cell suspension was centrifuged at ~200 × ***g*** for 5 min at room temperature and washed gently with advanced DMEM supplemeted with glutamax, HEPES and penicillin–streptomycin‐neomycin antibiotic Mixture (advDMEM +/+/+). Erythrocytes from the cell pellet were removed by washing with ammonium‐chloride‐potassium (ACK) lysis buffer (Life Technologies) for 2 min. Cell pellet were spun down at ~200 × ***g*** for 5 min and resuspended with advDMEM +/+/+ for cell counting and spun down again. Cell pellet was resuspended in 100% Matrigel (Corning) at approximately 1 × 10^3^ cells/μL Matrigel and seeded in a pre‐warmed 24‐well plate (Greiner Bio‐One). The plate was turned upside‐down to allow polymerization of Matrigel drop by incubating for 15 min at 37°C and 5% CO_2_. After the solid drops formed, 0.5 mL organoid culture medium was added to the well, the organoid cultures were maintained in a 37°C and 5% CO_2_ incubator and medium was changed every 3–4 days.

Typically, around 50%–80% of cell clusters would propagate to organoids within 2–3 weeks, therefore, organoids were passaged at a 1:2–3 dilution, for organoids which could not expand extensively, they would be passaged at a 1:1 dilution. For passaging, TrypLE express enzyme (Life Technologies) was added to digest the Matrigel. Dissociated the small cell clusters mechanically by pipetting followed by incubation for 3–5 min at 37°C. Cell suspension was spun down at ~200 × ***g*** for 5 min, cell pellet was resuspended in Matrigel. In order to stock cryopreserved lines, organoids were snap‐frozen in 90% FBS and 10% dimethyl sulfoxide (DMSO, Sigma‐Aldrich) and stored in liquid nitrogen. Frozen stocks could be utilized for recovery for downstream analysis.

### 2.3 Immunofluorescence, histology and immunohistochemistry

Organoids were fixed with 4% paraformaldehyde for 2 h permeabilizing with Triton X‐100 (BDH Laboratories) overnight. Unspecified bindings were blocked with 10%BSA for 2 h followed by incubation with primary antibodies overnight at 4°C. Incubated with goat anti‐mouse IgG‐FITC conjugated secondary antibody (Life Technologies) for 2 h followed by incubating in Hoechst staining for 2 h. The slides were mounted with coverslip and anti‐fade aqueous mountant (Life Technologies) and sealed with nail polish to prevent drying. The slides were viewed under confocal microscope and the images were analyzed and processed with Fiji software (v1.53q) (https://imagej.nih.gov/ij/).

Primary tissue organoid and ectopic xenograft were processed for paraffin sectioning using standards protocols. Organoids were fixed in 4% paraformaldehyde for 1 h. Organoids were embedded histogel (Thermo Scientific) before tissue processing and embedding. Paraffin blocks were sectioned at a 5 μm and performed hematoxylin–eosin staining using standard protocols.

Immunohistochemistry staining performed on 5 μm paraffin section using standard protocols. De‐paraffinization was performed in xylene followed by heat‐induced antigen retrieval in sodium citrate buffer (pH 6.0) or Tris‐EDTA buffer (pH 9.0). Slides were blocked in 2.5% normal horse serum (Vector) followed by incubating with primary antibodies overnight at 4°C. Slides were then incubated in HRP‐conjugated secondary antibody (Vector) at room temperature for 1 h before color development with DAB chromogen (Vector). Counterstaining with hematoxylin and dehydrating with ethanol and xylene were performed before mounting.

Histological examination, both HE and IHC, of primary tissue, organoid and xenograft were performed, thanks to pathologists from the Department of Anatomical and Cellular Pathology, CUHK, for the classification of tumor subtypes, pathological stages and histological grades according to AJCC 8th edition and WHO/ISUP or Fuhrman grading system.

### 2.4 Whole exome sequencing

Whole‐genome DNA from buffy coat, tumor tissue and organoid were extracted with QIAamp DNA mini kit or QIAamp DNA blood mini kit (Qiagen). Exome capture was performed by SureSelect Human All Exome V6 (58M) (Agilent Technologies). Sequencing will be performed on an Illumina platform (PE150) according to effective concentration of the library and data amount to be needed per sample (12G, 200X) by Novaseq 6000 instrument (Illumina). We applied BWA (v0.7.17) to align WES data to the human genome (hg19) with default settings. Mutect, strelka and platypus were employed to generate somatic mutations, insertions and deletions within each normal‐tumor sample pair. Mutations called by at least two programs were selected and manually inspected for further annotation analysis. The effect of somatic alterations was annotated and predicted with SnpEff. Somatic copy number alterations were identified by Control‐FREEC (v11.0) with a sliding window size of 5000 bp. Statistical comparisons and graphical representations were performed by “R” script and RStudio (v1.4) (https://www.rstudio.com/) with the “R” package maftools.

### 2.5 Organoid‐derived xenograft

Organoids were implanted subcutaneously to immunocompromised mice (6–8 weeks old). Organoid suspension with 1 × 10^5^ cells in 50% Matrigel/50% PBS was delivered into each subcutaneous site with a 25G needle 5/8‐inch needle. The size of subcutaneous tumor was measured by a caliper every 2 days for a maximum of 6 months. Immunocompromised mice were sacrificed and the subcutaneous tumor was harvested when a reduction of tumor size was observed in two consecutive measurements. Fresh tumor specimens were divided into two portions, fixation for IHC staining and frozen stock generation.

### 2.6 Sunitinib‐resistance cell line

Clear cell carcinoma cell line 786‐O (ATCC) was cultured in RPMI 1640 supplemented with 10% FBS at 37°C in a humidified incubator with 5% CO_2_. Culture medium was changed every 3–4 days. 786‐O was treated with sunitinib (Biorbyt) at 70–80% confluence sunitinib at an initial concentration of 0.5 μM. After the cells stabilized, passaging of cells was performed every 3–4 days. Concentration of sunitinib was increased stepwise by 0.5 μM to a final concentration of 15 μM to establish sunitinib‐resistant 786‐O‐R cells.

### 2.7 Drug screening assay

Tumor organoid, 786‐O or 786‐O‐R derived organoid cultures were digested with TrypLE to obtain single cells. Passed the cell aggregates through a 40 μm cell strainer, pelleted cells and resuspended with Matrigel at a density of 2500 cells/well of a 384 well plate. Organoids were allowed to proliferate for 3 days at 37°C in a humidified incubator with 5% CO_2_ followed by digestion with TrypLE and gently disrupted the Matrigel and resuspending in advDMEM +/+/+. Passed the cells through a 40 μm cell strainer and resuspended organoid in 2% Matrigel followed by dispensing organoids into Matrigel‐coated 384 well plate. Treat organoids with sunitinib (Biorbyt) and everolimus (MedChemExpress) (tumor organoids only) dose serial (0.1 μM to 100 μM), DMSO control and organoid medium control. Performed CellTiter‐Glo 3D Cell Viability Assay (Promega) within 6 days incubation upon drug administrations.

## 3 RESULTS

### 3.1 Clinical characteristics

A total of 78 patients, 52 males and 26 females, were recruited. The mean age of patients at diagnosis is 63.5 ± 9.34 (Figure S1). Twenty‐three patients had smoking history (8 current smokers and 15 ex‐smokers) while 48 of them were non‐smokers and 7 patients did not report their smoking status (Table 1).

**TABLE 1:** Summary of demographics and clinical characteristics of recruited patients.

Characteristics	Total Patients (*n* = 78)	
	*n*	*%*
Total age (years)		
20–40	2	2.5
41–60	23	29.5
61–80	53	68.0
Mean ± SD	63.5 ± 9.34	
Median (IQR)	66 (58,70)	
Range	32–79	
Age by gender (years)		
Male	64.3 ± 9.58^a^	
Female	61.8 ± 8.81^a^	
Gender		
Male	52	66.7
Female	26	33.3
Social history		
Current smoker	8	10.3
Ex‐smoker	15	19.2
Never smoker	55	70.5
Laterality		
Right kidney	45	57.7
Left kidney	33	42.3
Surgical intervention		
Partial nephrectomy	37	47.5
Radical nephrectomy	38	48.7
Others^b^	3	3.8
Tumor Subtypes		
ccRCC	63^c^	80.8
chRCC	4	5.2
pRCC	2	2.5
Unclassified	2	2.5
Sarcomatoid	1	1.3
Xp11 translocation	1	1.3
Succinate dehydrogenase‐deficient RCC	1	1.3
Adenocarcinoma	1	1.3
Adrenal tumor	1	1.3
Benign tumor^d^	2	2.5
Pathological stages		
pT1	51^e^	65.4
pT2	4	5.2
pT3	20	25.6
pT4	2	2.5
Benign	1	1.3
Histological grades		
Grade 1^f^	10	12.8
Grade 2^g^	43	55.2
Grade 3^h^	10	12.8
Grade 4^i^	6	7.7
Undetermined	9	11.5

^a^ Values were reported as mean ± S.D.

^b^ Three patients received adrenalectomy, abdominal excision and thrombectomy of IVC.

^c^ One patient was diagnosed with ccRCC and papillary adenoma.

^d^ Benign tumor includes renal abscess and oncocytoma.

^e^ One patient was diagnosed with benign oncocytoma.

^f^ Four patients were classified with Fuhrman grade.

^g^ Ten patients were classified with Fuhrman grade.

^h^ One patient was classified with Fuhrman grade.

^i^ One patient was classified with Fuhrman grade.

Regarding the tumor subtypes of RCC tumor, common and rare subtypes were observed in our cohort. Whereas ccRCC remains the dominant subtype, 63 patients were diagnosed with ccRCC, while 4 patients were diagnosed with chRCC and 2 patients were diagnosed with pRCC. Of note, a rare Xp11 translocation RCC was also observed. Remaining patients were diagnosed with other RCC subtypes, benign conditions or unclassified RCC. As for pathological stages (AJCC 8th edition), except undetermined in 14 patients, 43 patients were recognized as pT1 (pT1a and pT1b) diseases, 3 were diagnosed of pT2 diseases, 17 were identified as pT3 (pT3a, pT3b and pT3c) RCC and 1 patient was at pT4. Among all patients, 9 patients diagnosed with mRCC, 2 patients had local relapses and 1 were diagnosed with isolated recurrence in infrarenal inferior vena cava (IVC). Majority of patients were classified to different histological grades by WHO/ISUP grading system, though 16 patients were determined with Fuhrman grades. Low grade (Grade 1–2) tumors were observed in 10 and 43 patients respectively while high grade tumors (Grade 3–4) were noticed in 10 and 6 patients respectively, whereas 9 patients were neither graded by WHO/ISUP nor Fuhrman grades (Figure S2A–E).

### 3.2 Tumor organoids were differentiated from normal organoids and recapitulated histopathological features of parental RCC tumor

**FIGURE 2:**
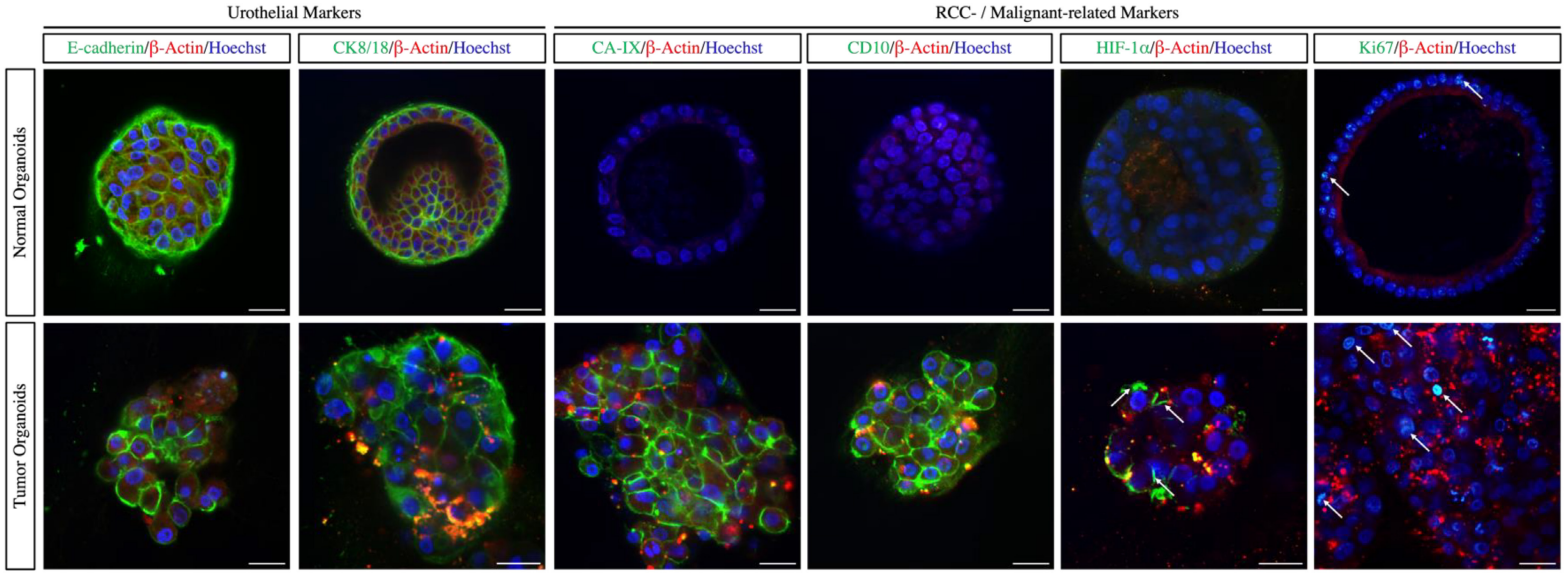
Immunofluorescent images of normal and tumor organoids with different markers. Normal organoids could be morphologically differentiated from tumor organoids as the former exhibited a regular structure while latter demonstrated an irregular architecture. Both normal and tumor organoids expressed urothelial markers while only tumor organoids expressed cancer markers. The levels of Ki67 was higher in tumor than normal organoids.

Confocal imaging highlighted the presence of a differentiated population expressing CK8/18 and E‐cadherin, two typical markers of the urinary tract epithelium at the periphery of 3D‐cultures and both normal and tumor organoids exhibited membranous expression patterns. In addition, normal organoids did not exhibit expressions toward CA‐IX and CD10, which are RCC‐specific markers, while organoids derived from ccRCC exhibited membranous expression patterns. HIF‐1α was stabilized as a result of *VHL* inactivation and it was strictly associated to RCC, therefore, HIF‐1α expression was observed only in tumor organoids. Besides, we also detected Ki67, which indicated proliferation and the presence of actively dividing cells, in both normal and tumor organoids. Nonetheless, population of organizing aggregates exhibited positive Ki67 stain in tumor organoids were higher than that of normal organoids, suggesting the tumor organoids were better in maintenance and propagation than normal organoids under similar passage (Figure 2).

**FIGURE 3:**
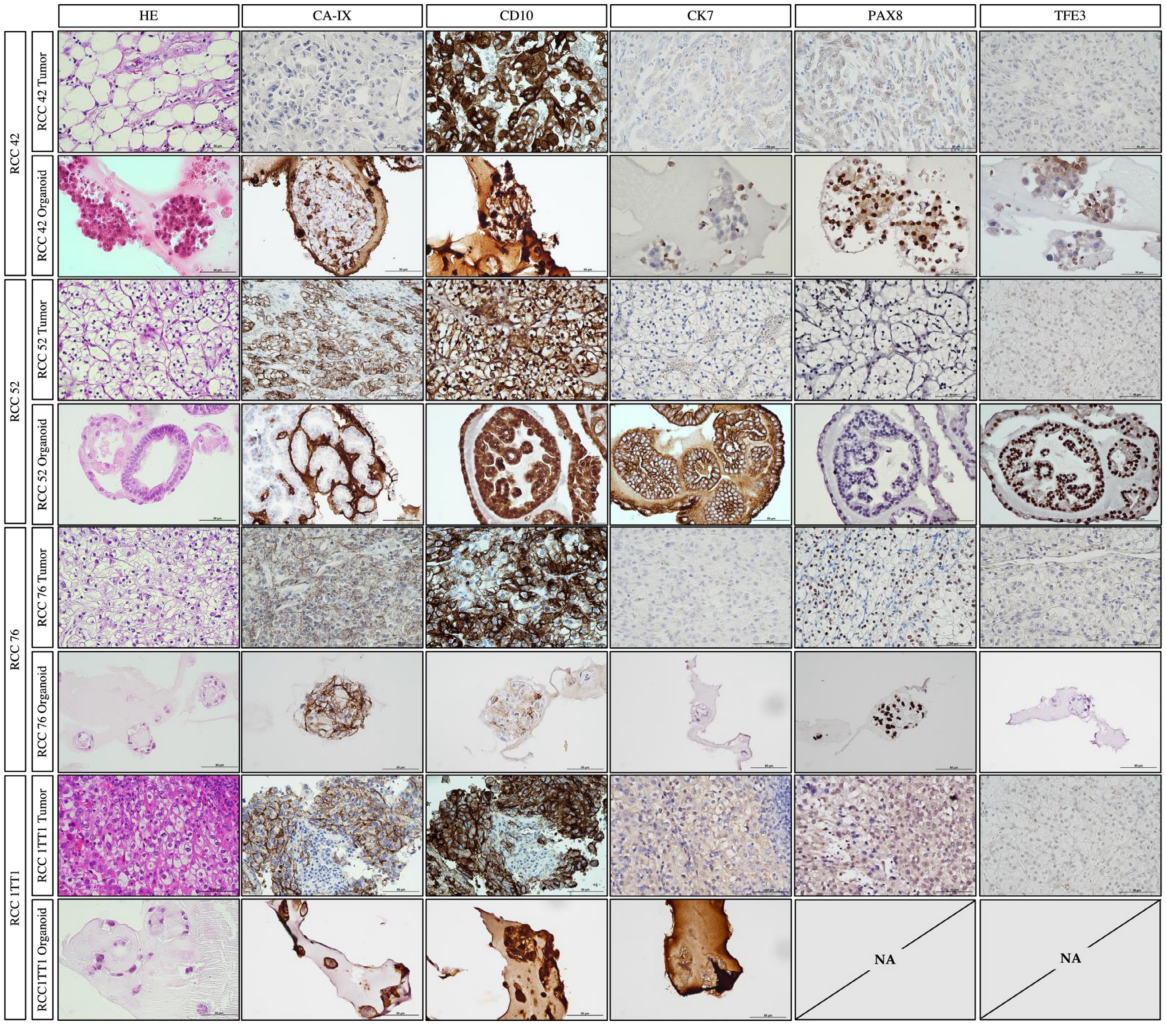
Representative IHC images of parental tumor tissue and derived organoid lines. IHC staining indicated that high degree of association of immunoexpressions were generally observed between tumor and organoids. Tumor IHC images were at 20×, scale bar indicated 50 μm. Organoid IHC images were at 40×, scale bar indicated 50 μm.

**FIGURE 4:**
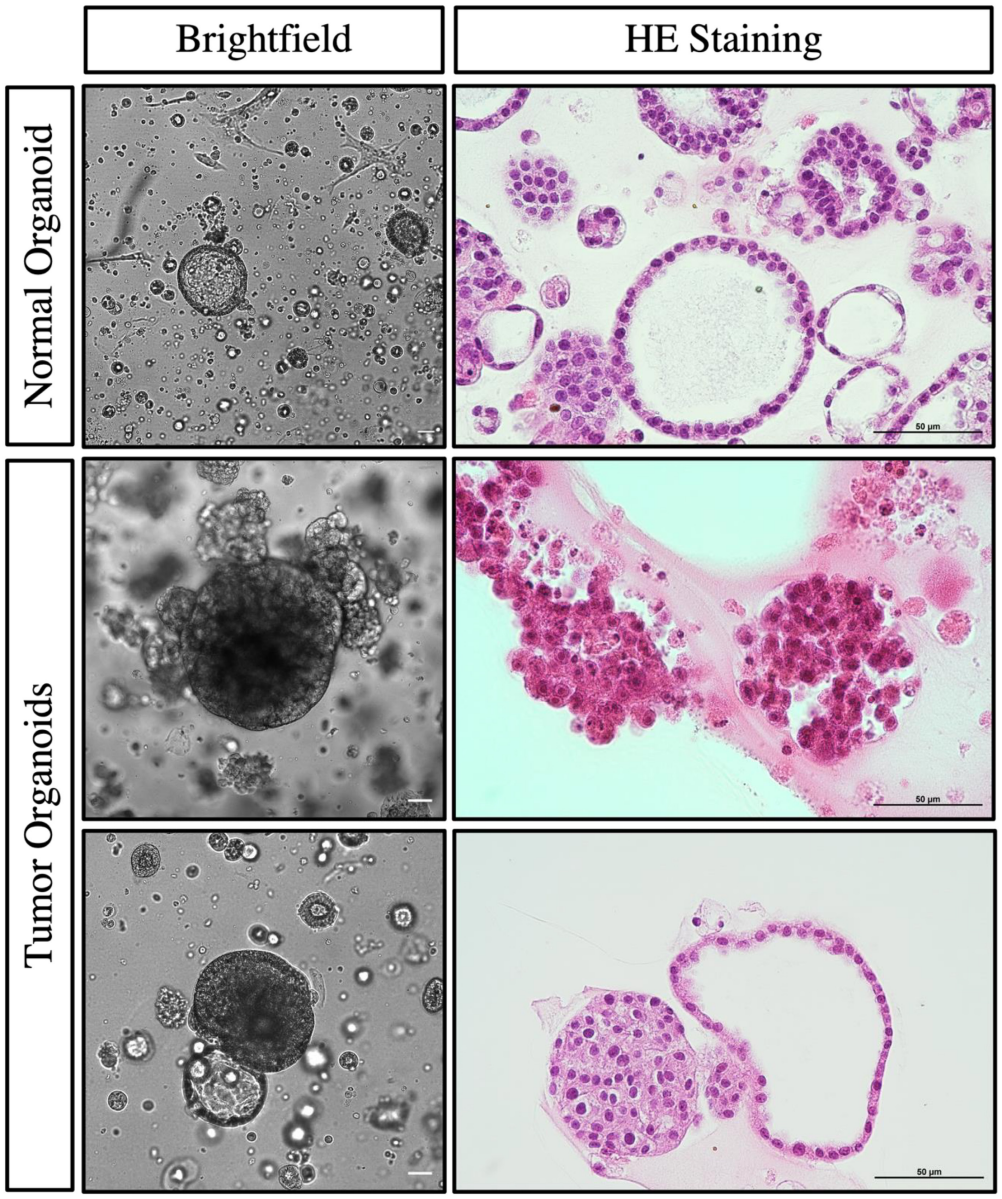
Brightfield and HE staining images of normal organoid and tumor organoid cultures. Normal organoid cultures exhibited a clear lumen while tumor organoid cultures exhibited cell aggregates or mixed morphologies. Brightfield images were at 10×, scale bar indicated 50 μm. HE staining images were at 40×, scale bar indicated 50 μm.

We observed concordance in immunoexpressions between RCC tumors and their corresponding organoids (Figure 3), nonetheless, some expression patterns are observed in organoids, but not the parental tumor tissues. For example, CK7 was observed in RCC52 organoids but not in its parental tumor tissues. Besides, we observed morphological differences between normal and tumor organoids. Majority of normal organoids exhibited a clear lumen with cells lining in the peripheral revealed by HE staining. While tumor organoids grew in dense clusters of cells, forming aggregates and clumps. Besides, cells in tumor organoids arranged irregularly and composing a loosely organized 3D structures of different sizes. Nonetheless, tumor organoids conceivably comprised of non‐malignant populations, therefore, mixed morphologies were observed sometimes (Figure 4).

### 3.3 Organoid lines recapitulated mutational spectrum of human RCC

**FIGURE 5:**
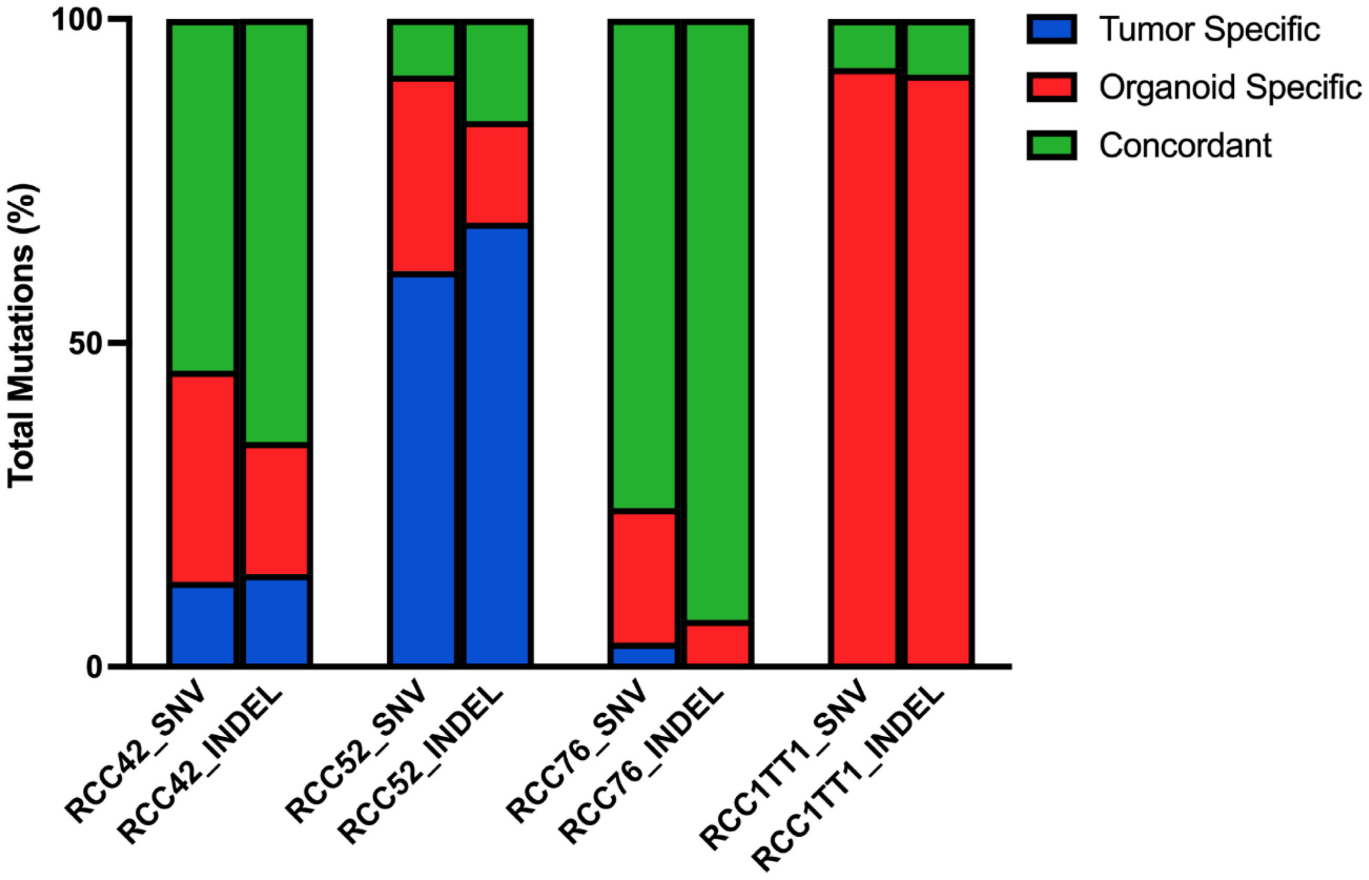
Concordance of mutations detected in RCC42, RCC52, RCC76 and RCC1TT1 parental tumor and organoid in terms of SNV and INDEL.

**FIGURE 6:**
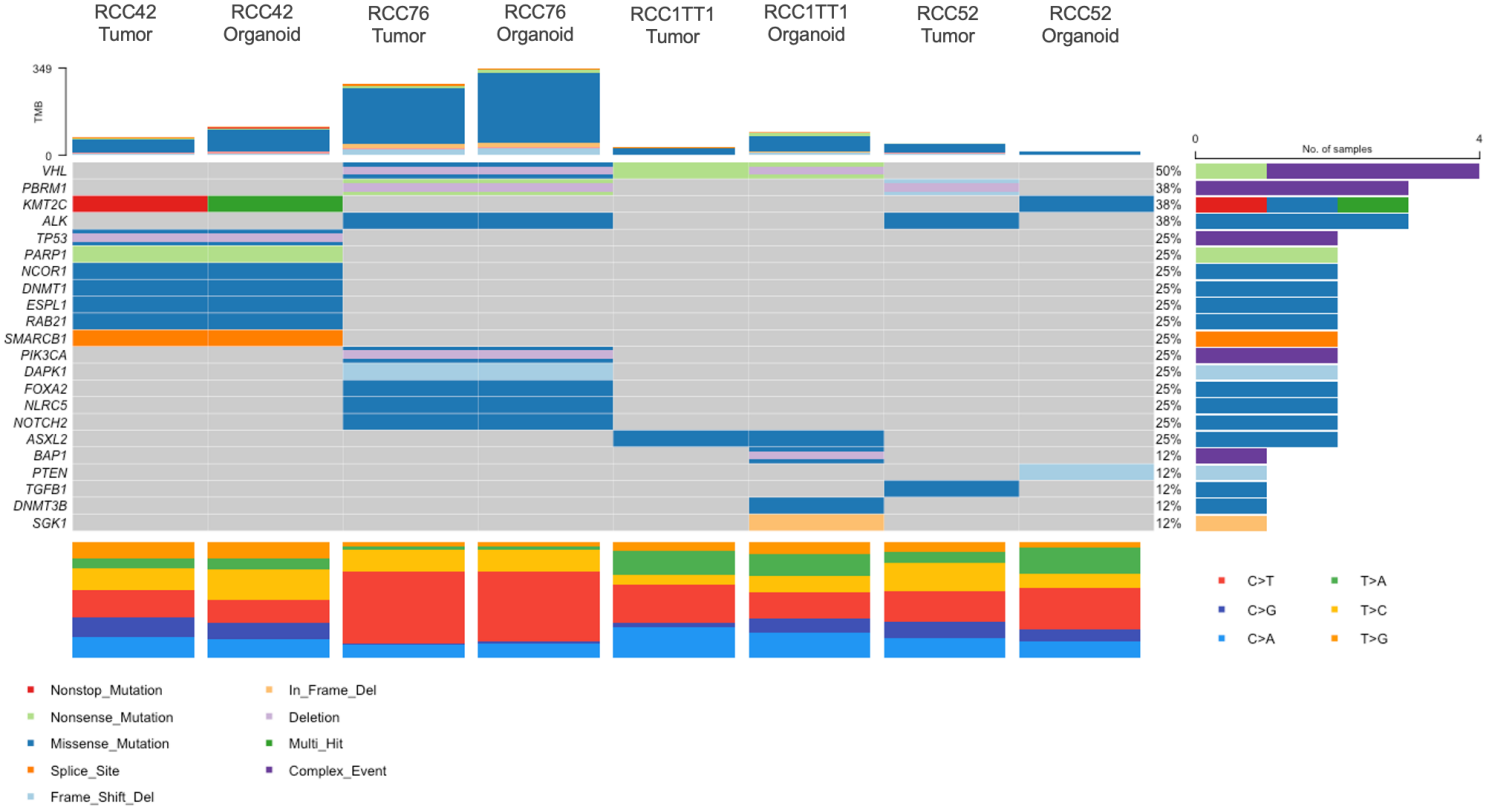
Mutation landscape of RCC organoids. Somatic mutations and copy number alterations identified in organoids and parental tumors with whole exome sequencing. Representative genes were selected from TCGA database, and they were known to be mutated in kidney cancer.

Results from WES of four organoid lines (RCC42, RCC52, RCC76, and RCC1TT1) indicated the cellularity of parental tumor tissue was ranging from less than 20% to around 60% while overall concordance between derived organoids and primary tumor was around 10%–80%, (Figure 5). Variant data suggested that 4 of the cancer organoids and respective parental tumor tissue, exhibited mutations in common RCC drivers, RCC pathological‐related genes and cancer drivers, such as *VHL*, *PBRM1*, *KMT2C*, *BAP1*, *PIK3CA* and *TP53* (Figure 6). In addition, copy number alteration (CNA) analysis revealed a high similarity among parental and derived organoids (Figure S3–S6). Of note, from RCC42, three deep deletions, *UGT2B17*, *OR4P4* and *CES1P1* were consistently observed in both parental tumor and organoid lines. On the other hand, RCC52 organoid lines exhibit organoid‐specific mutations in *KMT2C* and *PTEN* were observed and confirmed by Integrative Genomics Viewer (IGV) inspection (Figure S7). In addition, RCC1TT1 organoid line's primary tumor was found to have a low tumor cellularity <20%. In view of that, no CNA from primary tumor could be identified while organoid lines exhibited a more prominent CNA. Since the cancer organoids recapitulated the mutations in cancer drivers and the chromosomal deletions, our cancer organoid models could represent the primary tumor tissue genetically.

### 3.4 Tumorigenesis in organoid‐derived xenografts

**FIGURE 7:**
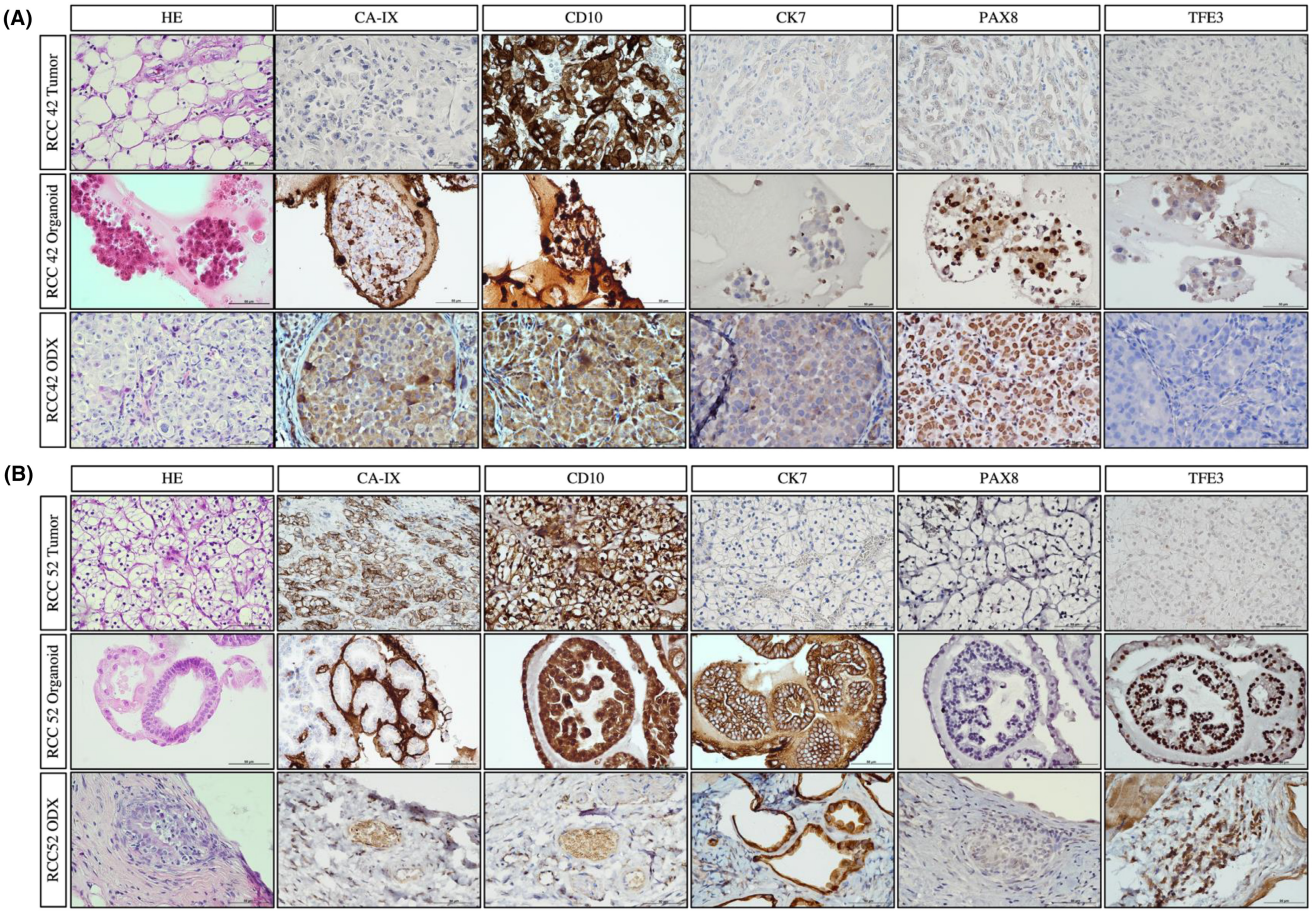
In vivo validation of tumorigenic ability of organoids in ectopic xenografts. (A) HE and IHC images of RCC42 parental tumor, organoid and ODX. IHC results were comparable between organoid lines and tumors from xenograft, indicating that the tumor was possibly derived from organoids. CA‐IX and PAX8 expressions were specific to organoid and ODX only. TFE3 should exhibit as nuclear stain, therefore, that of organoid was regarded as negative. (B) HE and IHC images of RCC52 parental tumor, organoid and ODX. IHC results were comparable between organoid lines and tumors from xenograft, indicating that the tumor was possibly derived from organoids. CK7 and TFE3 expressions were specific to organoid and ODX only. HE and IHC images were at 40×, scale bar indicated 50 μm.

Cancer organoids were selected for tumorigenesis only after genetic validations through WES. As there was an inadequate number of cells from RCC1TT1 and RCC52 due to a low doubling time, we have selected two cases (RCC42 and RCC52) for xenograft establishment. Tumors were observed from both RCC52 and RCC42 derived xenograft (Figure S8). We have challenged the tumors with a panel of IHC markers (CA‐IX, CD10, PAX8, CK7, and TFE3). We observed that the immunoexpression patterns of RCC52‐derived ODX were comparable with RCC52 organoid lines. Intriguingly, both organoid line and ODX exhibited CK7 expression which was absence in parental tumor tissues. In addition, CA‐IX and CD10, which are markers of ccRCC as well as TFE3 were also stained positive in both organoid lines and ODX. Nonetheless, PAX8 was not detected in organoid lines and ODX. Another xenograft model generated by RCC42 organoid line also exhibited high similarities in immunoexpression pattern. Results of IHC on CD10, CK7 and TFE3 were concordant among parental tumor tissues, organoids and ODX, while PAX8 and CA‐IX expressions were specific to organoid and ODX only (Figure 7A,B). Therefore, tumor growth was observed after cancer organoids inoculation into immunocompromised mice. The harvested tumor expressed similar markers when compared to cancer organoid and parental tumor tissues.

### 3.5 Organoid lines served as a platform for drug screening

**FIGURE 8:**
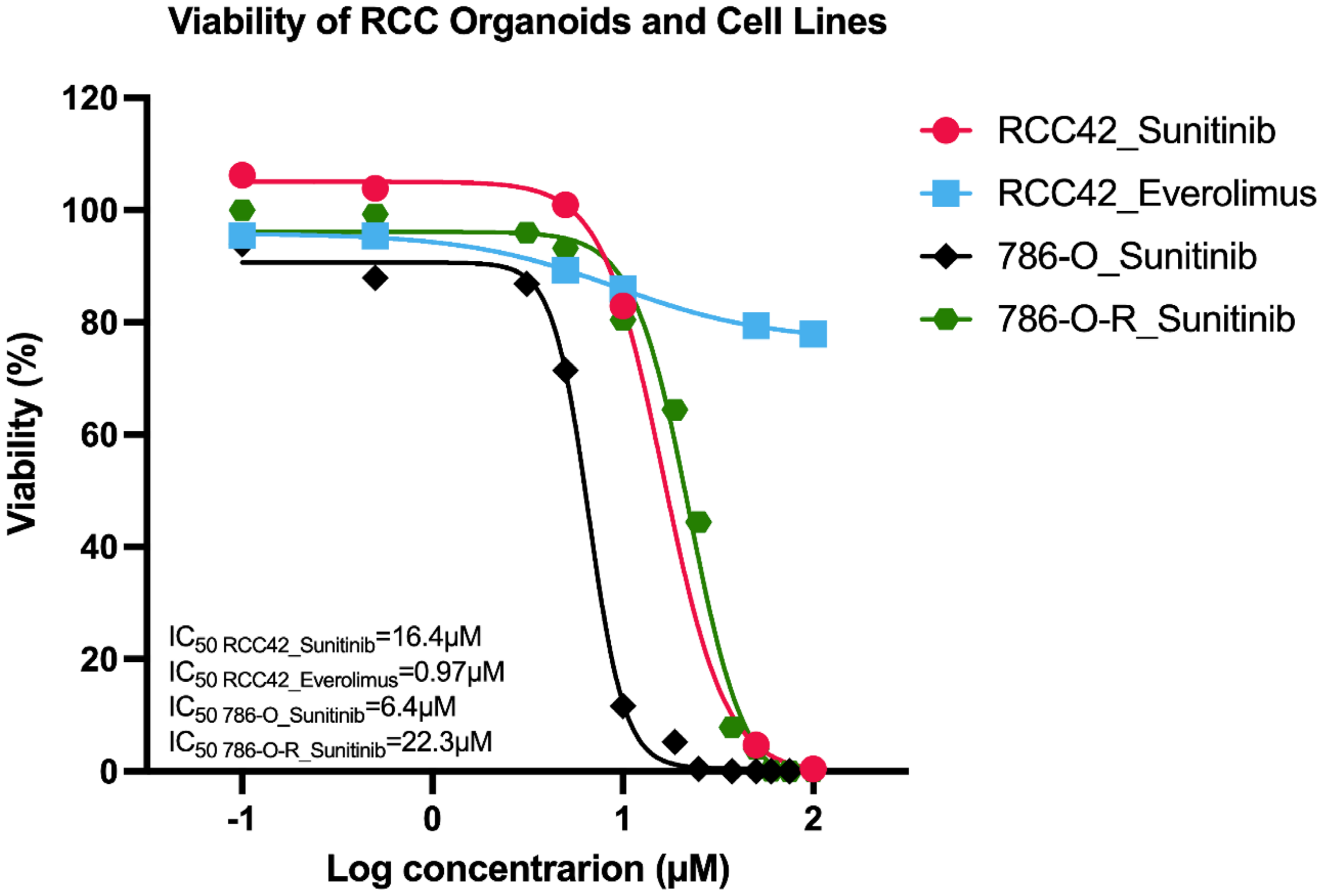
Dose response curve of RCC42 organoid lines and ccRCC cell lines upon indicated drug administration. 786‐O denotes sunitinib‐sensitive condition while 786‐O‐R denotes sunitinib‐resistant condition.

One of the above confirmed organoid lines (RCC42) was further challenged with Sunitinib and Everolimus. The patient (RCC42) was clinically given 800 mg of pazopanib initially. The patient then developed hand foot syndrome (skin problem) after drug administration, therefore, the dose was reduced to 400 mg. Notwithstanding, recurrent lesion was observed in surgical bed and metastatic lesion in brain were observed radiologically after that. Our organoid line results indicated that sunitinib inhibited proliferation of organoids and IC_50_ was identified at 16 μM. Intriguingly, Everolimus did not effectively attenuate proliferation of organoids, in fact, upon administration of everolimus, the organoid lines did not exhibit drastic change in viability. To further investigate whether RCC42 tumor organoids achieved resistance to sunitinib in vitro, we employed a ccRCC cell line 786‐O and sunitinib‐resistance ccRCC cell line 786‐O‐R for the analysis of viability upon sunitinib administration. 786‐O displayed a reduction in proliferation at a lower concentration, IC_50_ = 6 μM, than RCC42 organoids while 786‐O‐R cell line‐derived organoids exhibited resistance upon administration of low concentration of sunitinib. Nonetheless, viability was drastically diminished at high dose of sunitinib while IC_50_ was identified at 22 μM (Figure 8). Our cancer organoid model can potentially serve as a platform for drug screening, nonetheless, further experiments should be performed before translating the results in clinical settings.

## 4 DISCUSSION

We have validated our organoid model with IHC staining, results indicated that immunoexpression patterns of organoid lines were comparable with respective parental tumor tissues. Nonetheless, we noticed that expression patterns were not comprehensively concordant among all organoid cultures. Hereby, we suggested that small population expressed differently as parental tumor tissues maybe selected or more adapted to culture conditions upon the prolong culturing period.

We noticed our organoid lines harbored hallmark oncogenic events of RCC, including mutations in *VHL*, *PBRM1*, *PIK3CA*, and *BAP1*. Numerous studies had provided robust evidence and gave verdicts on the involvement of these genes in prognosis, development, maintenance and progression of RCC as well as the responses to treatments.^22^ Intriguingly, we observed somatic mutations in *BAP1* and *ASXL2*, which were involved in *BAP1*/*ASXL2* axis, in RCC1TT1 line. Studies identified *BAP1* as a tumor suppressor in ccRCC and was associated with aggressiveness, high‐grade nuclear features, metastasis.^23^, ^24^

Among our organoid lines, RCC42 displayed a *TP53* missense mutation. It is unequivocal that p53 is mutated in 50% human cancers. Nonetheless, expression of *p53* was only in one‐fifth of primary RCC, albeit more frequent in mRCC.^25^ This suggested that *p53* expression may be a relatively late event in the evolution of RCC and might associated with metastatic capabilities.^26^, ^27^ Strikingly, RCC42 patient endured a brain metastasis half year after resection of primary tumor. We suspected the mutation in *TP53* has made consensus with published studies.^28^, ^29^

Organoid lines also exhibited somatic mutations in RCC‐related genes which were in concordant with respective tumor including *ESPL1*, *SMARCB1*, *RAB21*, *NCOR1, NLRC5*, *NOTCH2*, and *FOXA2* mutations. There were studies indicated that all the forementioned genes were related with development and progression of RCC as well as pathological stages and histopathological.^30^, ^31^, ^32^, ^33^, ^34^, ^35^, ^36^

We observed mutation in drug target genes which implied possibilities in employing our model in drug screening and testing, including *PAPR1*, *DNMT1/3B*, *DAPK*, and *SGK1*. Several studies investigated the use of their inhibitors as novel target therapeutic agents. For example, PARP1 inhibitor (PARPi) was shown effective in significantly suppressing survival, proliferation, clonogenic ability in vitro and in vivo.^37^, ^38^ Besides, DNMT inhibitor (DNMTi) 5‐Aza‐2′‐deoxycytidine (5‐Aza‐CdR) has been employed, both in solitary and in synergism with chemotherapies, in pre‐clinical and clinical trials in urological malignancy setup, such as bladder cancer, prostate cancer and testicular cancer, in addition to their approved ground in treatment of hematological malignancies.^39^ It was shown that 5‐Aza‐CdR exhibited anti‐tumoral effects in vitro and in vivo.^39^, ^40^ The advantages of organoids could be applied in choosing the suitable and precise therapeutic agents for the patients.^37^, ^38^, ^40^, ^41^, ^42^, ^43^ In addition, *DAPK* was correlated with sunitinib sensitivities while *SGK1* was found associated with IL‐2 responsiveness.^44^, ^45^ This raised the concerns for the effectiveness and safety of the immunotherapy in RCC patients, in fact, aberrations in anti‐tumoral pathways might trigger the immune escape from effective treatment and thereby favored the survival of cancer populations.^46^

We hereby also presented a xenograft model which recapitulated immunoexpression patterns of organoids. Especially for RCC52, WES results did not robustly support RCC52 organoids were originated from parental tumor tissues, an organoid specific *PTEN* deletion was observed, nonetheless. We suspected early malignant population was elected and amplified in organoids which preserved the tumorigenesis ability in vivo. Remarkedly, both organoid lines and tumor harvested from ODX consistently displayed a heterogenous expression of CK7 as the parental tumor tissues. This further provided evidence that the harvested ODX tumor was derived from organoid lines.

We demonstrated that our RCC organoid model could potentially serve as a platform in choosing suitable drug interventions for patients and we sought to provide correlations between our in vitro results and clinical observations. In fact, sunitinib and pazopanib were among the most frequently adopted TKI in mRCC which inhibit VEGF and VEGFR and had similar efficacies.^47^, ^48^, ^49^, ^50^ IC_50_ of organoid growth was higher than the clinically relevant concentration (i.e., peak plasma concentration) of sunitinib (200 nM).^51^ Therefore, the organoids were considered as sunitinib‐resistance in clinical setup though the patient received pazopanib. In addition, we sought to evaluate whether RCC42 organoid lines demonstrated sunitinib resistance in vitro. In this study, we regarded 786‐O as a generalized model for to represent treatment naïve patients who are sensitive to sunitinib while 786‐O‐R mimicked sunitinib‐resistant condition ex vivo. Cell viabilities results demonstrated that 786‐O displayed attenuated proliferation at a lower dose of sunitinib than RCC42 organoids and 786‐O‐R. As IC_50_ of sunitinib from RCC42 and 786‐O‐R was comparable, this gave us evidence to conclude RCC42 achieved sunitinib resistance in vitro.

On the other hand, the organoid line showed resistance to everolimus as proliferation of organoids was almost unaffected. The results primed us intriguing insights. Everolimus functions as a mTOR inhibitor (mTORi) that targeted mammalian mTOR serine–threonine kinase.^52^ It was approved for second‐ and third‐line therapy in patients with advanced RCC.^53^, ^54^ Our model presented that everolimus might not be a suitable choice of second‐line treatment for the RCC42 patient as growth inhibition effect was not explicitly observed.

Altogether, organoid lines exhibited mutations in drug target genes which allowed the possibility to apply the model in drug screening and the development of target therapy. Besides, RCC organoids also demonstrated high concordance in somatic mutation of genes involving in cancer RCC development, maintenance and progression. Therefore, RCC organoids can serve as a platform for disease modeling and characterization of cancer subtypes.

## 5 LIMITATIONS

Our current study was not without limitations. In the first place, contamination of non‐malignant cells was prominent in our cultures. Results from tumor cellularity, copy number, somatic mutation, tumor cellularity and Sanger sequencing analyses collectively primed us to the suspicion that current culture condition elected predominately benign population. In addition, several factors affected the successful rate of organoid derivation. Tumor qualities such as severe necrosis and presence of adipose tissues hindered our model establishment.^55^ In addition, undifferentiated tumor populations with lose contact inhibition and differentiation capacity might acquire invasive properties and fall to create 3D‐structure.^55^

Culture conditions including but not limited to organoid medium were critical in establishing a successful model. We have not formulated the most optimal conditions suggested by the low successful rates. Several studies demonstrated that maintaining stemness of tumor cells were critical to the growth of tumor organoids.^56^, ^57^, ^58^ Thereby, growth factors or supplements that interact, either passively or directly, on stemness regulators should be augmented to current culture media.

Collectively, eliminating non‐malignant population and modifying culture medium should be performed in future studies in order to address the limitations and obstacles of current study.

## 6 CONCLUSION

In this study, we validate our organoid models in vitro and in vivo, organoids demonstrated similar immunoexpressional patterns as compared with parental tumor tissues. In addition, tumorigenesis was also observed in vivo. Validation from WES was considered as most important when comparing concordance in genetics between primary tumor and derived organoids. We observed concordant mutations in driver genes, histopathological genes and drug target genes. Lastly, drug screening assay provided promising results in application of our RCC model. In a nutshell, our current model gave promising results for disease modeling in RCC and served as a hypothetical platform in drug screening.

## AUTHOR CONTRIBUTIONS

**Ryan Tsz‐Hei Tse:** Conceptualization (equal); formal analysis (equal); investigation (equal); methodology (equal); writing – original draft (equal); writing – review and editing (equal). **Christine Yim‐Ping Wong:** Conceptualization (equal); investigation (equal); methodology (equal); supervision (equal); validation (equal); writing – original draft (equal); writing – review and editing (equal). **Xiaofan Ding:** Formal analysis (equal); software (equal); visualization (equal). **Carol Ka‐Lo Cheng:** Investigation (equal); methodology (equal); writing – review and editing (equal). **Chit Chow:** Conceptualization (equal); methodology (equal); resources (equal). **Ronald Cheong‐Kin Chan:** Conceptualization (equal); methodology (equal); resources (equal). **Joshua Hoi‐Yan Ng:** Investigation (equal); resources (equal). **Victor Wai‐Lun Tang:** Investigation (equal); resources (equal). **Peter Ka‐Fung Chiu:** Project administration (equal); resources (equal); supervision (equal); writing – review and editing (equal). **Jeremy Yuen‐Chun Teoh:** Conceptualization (equal); project administration (equal); resources (equal); writing – review and editing (equal). **Nathalie Wong:** Methodology (equal); supervision (equal). **Ka‐Fai To:** Funding acquisition (equal); project administration (equal); supervision (equal). **Chi‐Fai Ng:** Conceptualization (equal); funding acquisition (equal); project administration (equal); resources (equal); supervision (equal); writing – review and editing (equal).

## FUNDING INFORMATION

This study was funded by the CUHK Direct grant 2018.098.

## CONFLICT OF INTEREST STATEMENT

The authors declare that the research was conducted in the absence of any commercial or financial relationships that could be construed as a potential conflict of interest.

## ETHICS STATEMENT

This study was reviewed and approved by The Joint Chinese University of Hong Kong–New Territories East Cluster Clinical Research Ethics Committee. The patients/participants provided their written informed consent to participate in this study.

## Supporting information


Figure S1.

## Data Availability

The datasets presented in this study can be found in online repositories. The names of the repository/repositories and accession number(s) can be found in the article/ supplementary material.
