# Supplementary material for: The establishment of kidney cancer organoid line in drug testing
Source: Cancer Med. 2024 Jun 26;13(12):e7432. doi: 10.1002/cam4.7432 (PMC11200131; doi:10.1002/cam4.7432)
Supplement: Supplementary file 1 — Figure S1. [file CAM4-13-e7432-s001.pdf]

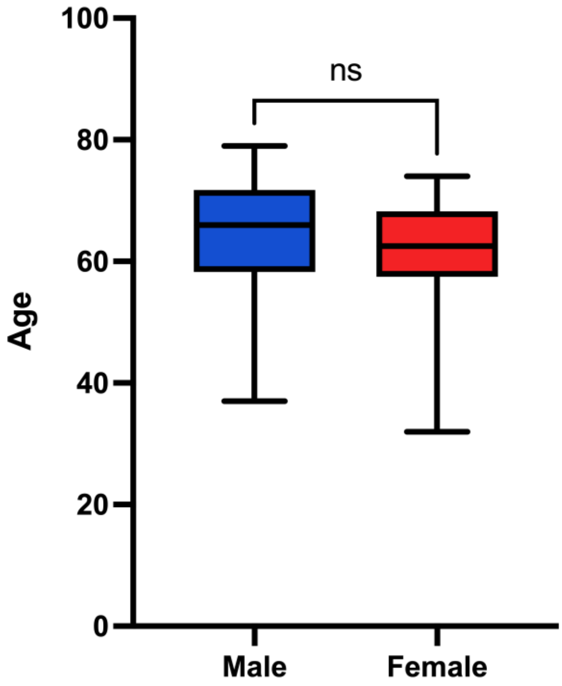

Figure S1. Representation of mean age at diagnosis across genders. No significance (*p*=0.261) of the age at diagnosis was observed between males and females.

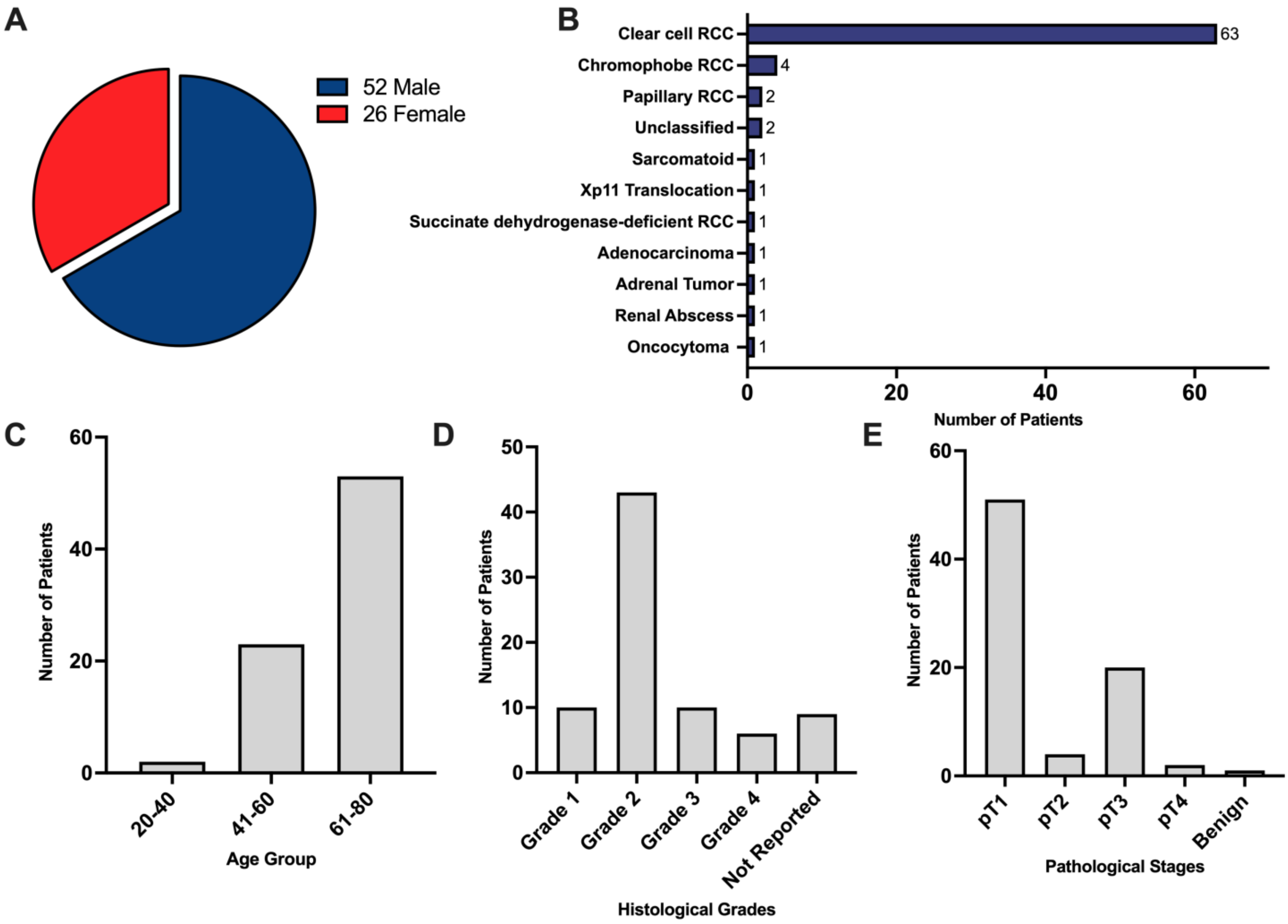

Figure S2. Graphic illustration of major clinical characteristics of recruited patient cohort. (A) Gender distribution of patient cohort. (B) RCC tumor subtypes. (C) Age distribution of patient cohort. (D) Histological grades classified by WHO/ISUP or Fuhrman grading. (E) Pathological stages of RCC tumors.

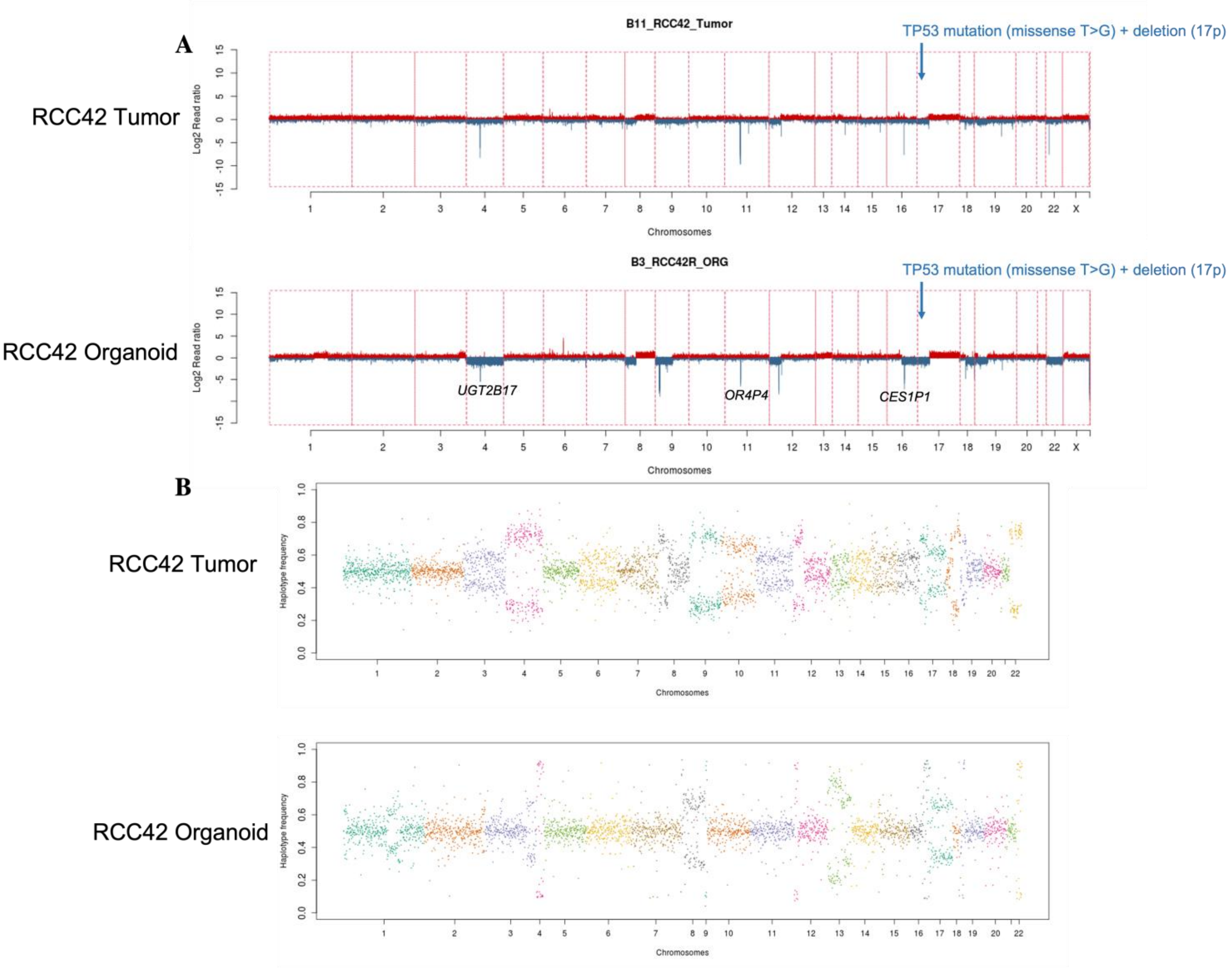

Figure S3. Copy number alternations identified from RCC42 organoid lines and parental tumor by whole exome sequencing. (A) Log2 ratio representation of CNA, *TP53* deletion at 17p was observed concordantly from organoids and parental tumor. 3 deep deletions, *UGT2B17*, *OR4P4* and *CES1P1* were preserved in organoids. (B) B-allelic frequency representation of CNA, patterns of chromosomal deletion between organoids and parental tumor were comparable.

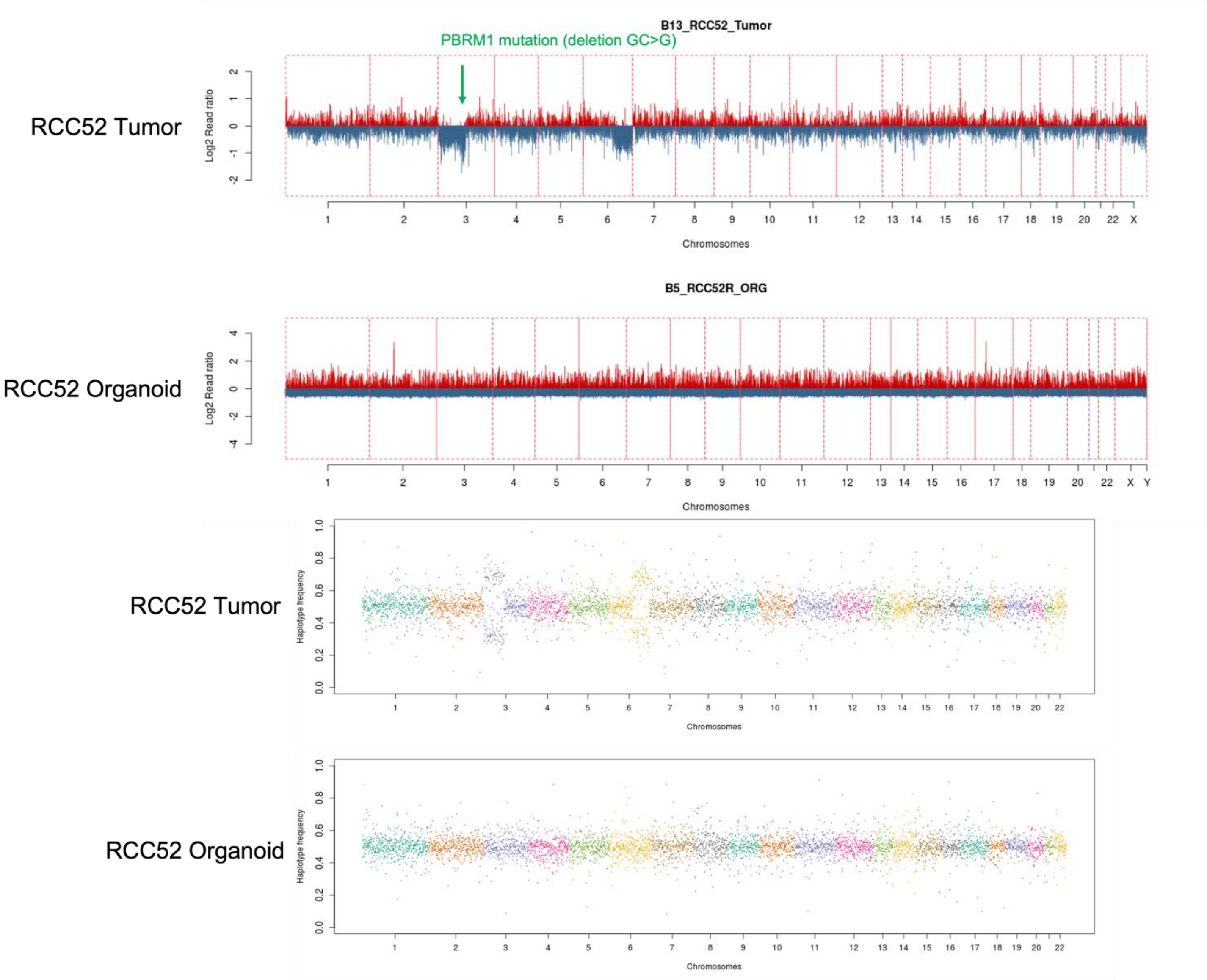

Figure S4. Copy number alternations identified from RCC52 organoid lines and parental tumor by whole exome sequencing. (A) Log2 ratio representation of CNA, *PBRM1* deletion at 3p was only from parental tumor whilst derived organoids did not exhibit major chromosomal aberrations. (B) B-allelic frequency representation of CNA, patterns of chromosomal deletion in primary tumor were distinguishable from derived organoids.

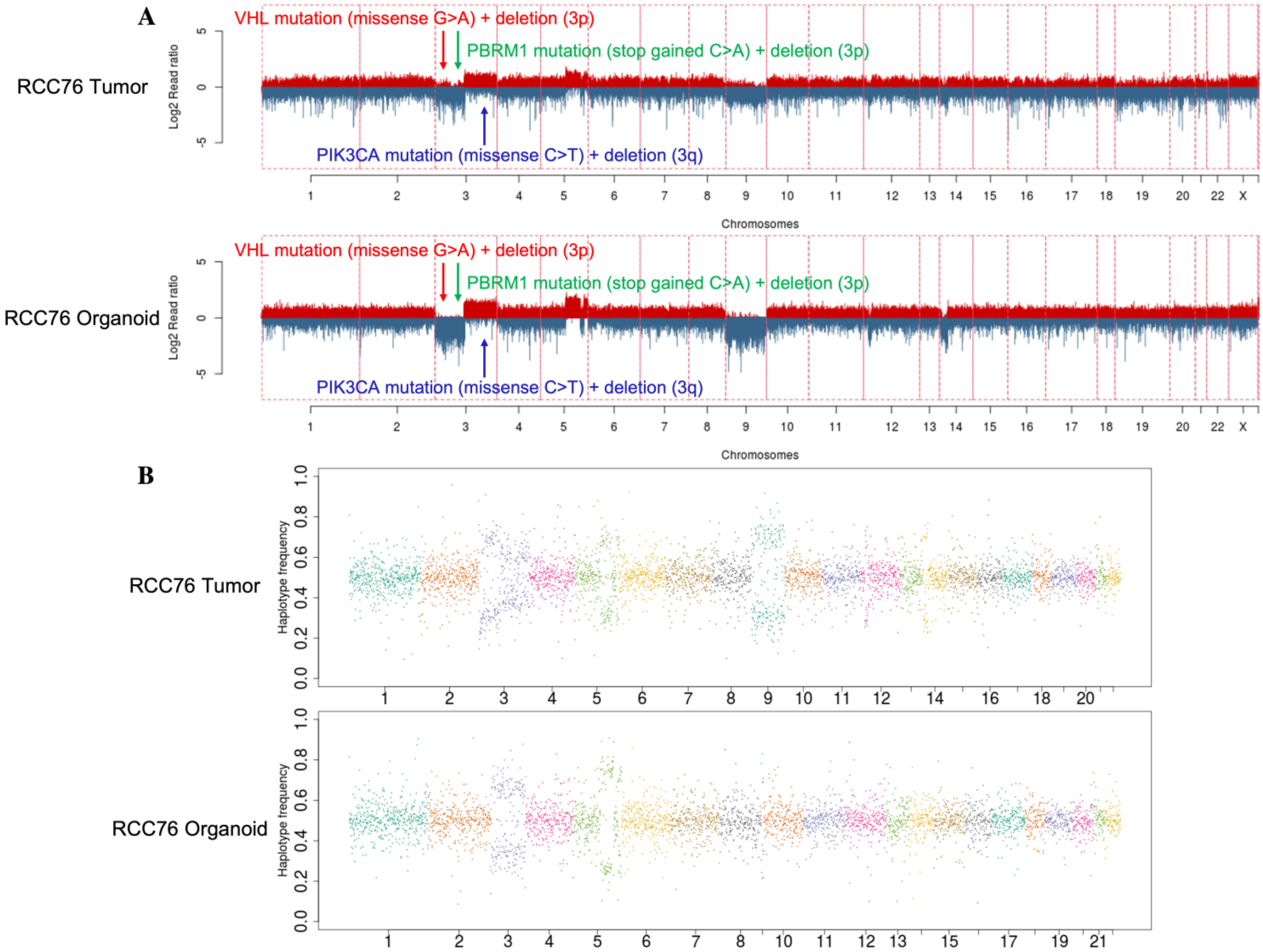

Figure S5. Copy number alternations identified from RCC76 organoid lines and parental tumor by whole exome sequencing. (A) Log2 ratio representation of CNA, *VHL* and *PBRM1* deletions at 3p and *PIK3CA* deletion at 3q were observed concordantly from organoids and parental tumor. (B) B-allelic frequency representation of CNA, patterns of chromosomal deletion between organoids and parental tumor were comparable.

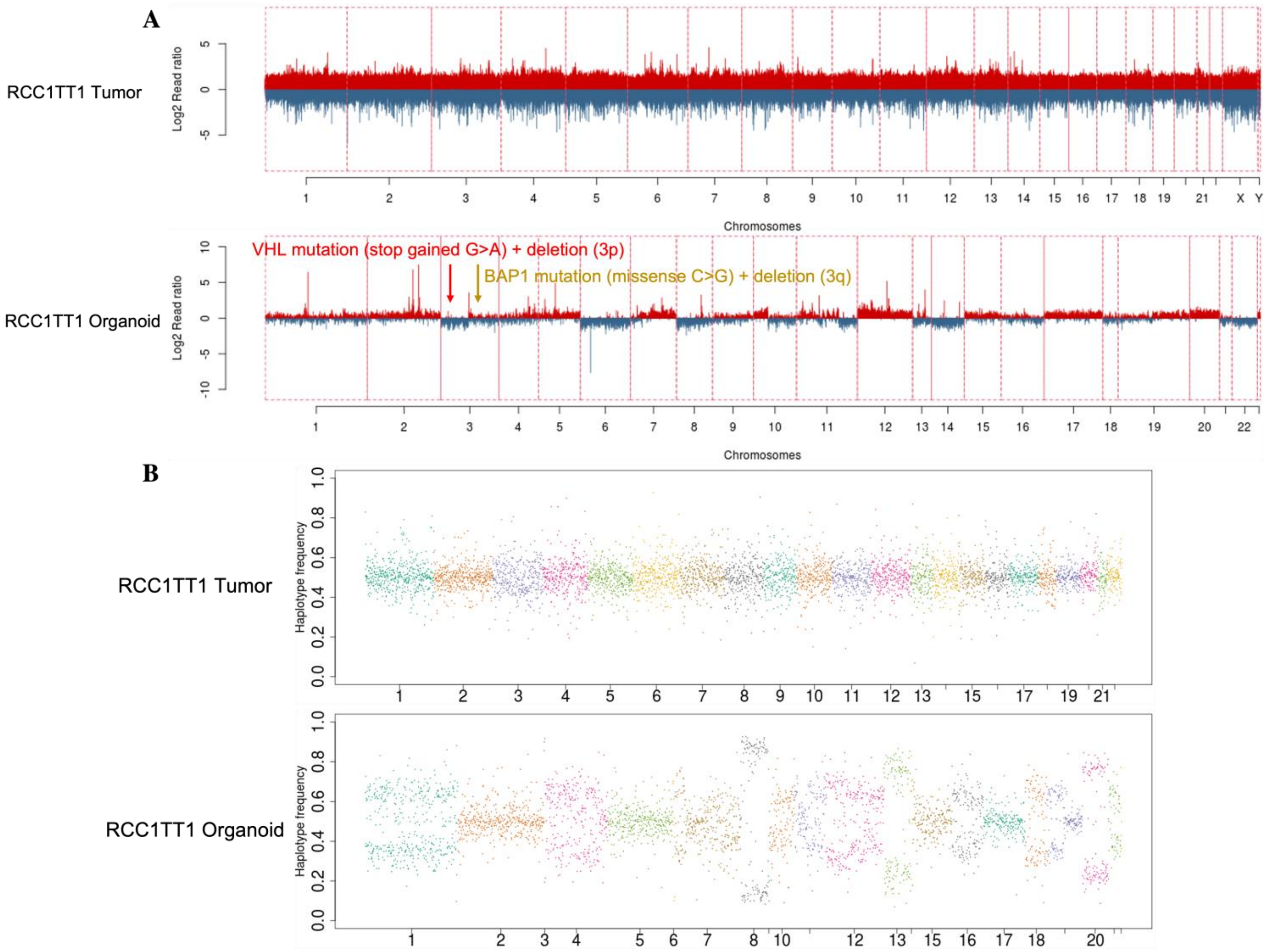

Figure S6. Copy number alternations identified from RCC1TT1 organoid lines and parental tumor by whole exome sequencing. (A) Log2 ratio representation of CNA, *VHL* deletion at 3p and *BAP1* deletion at 3q were observed only from organoids. (B) B-allelic frequency representation of CNA, patterns of chromosomal deletion in derived organoids were distinguishable from parental tumor. Due to low tumor cellularity of parental tumor, CNA could hardly be identified. Therefore, chromosomal aberrations could not be presented graphically.

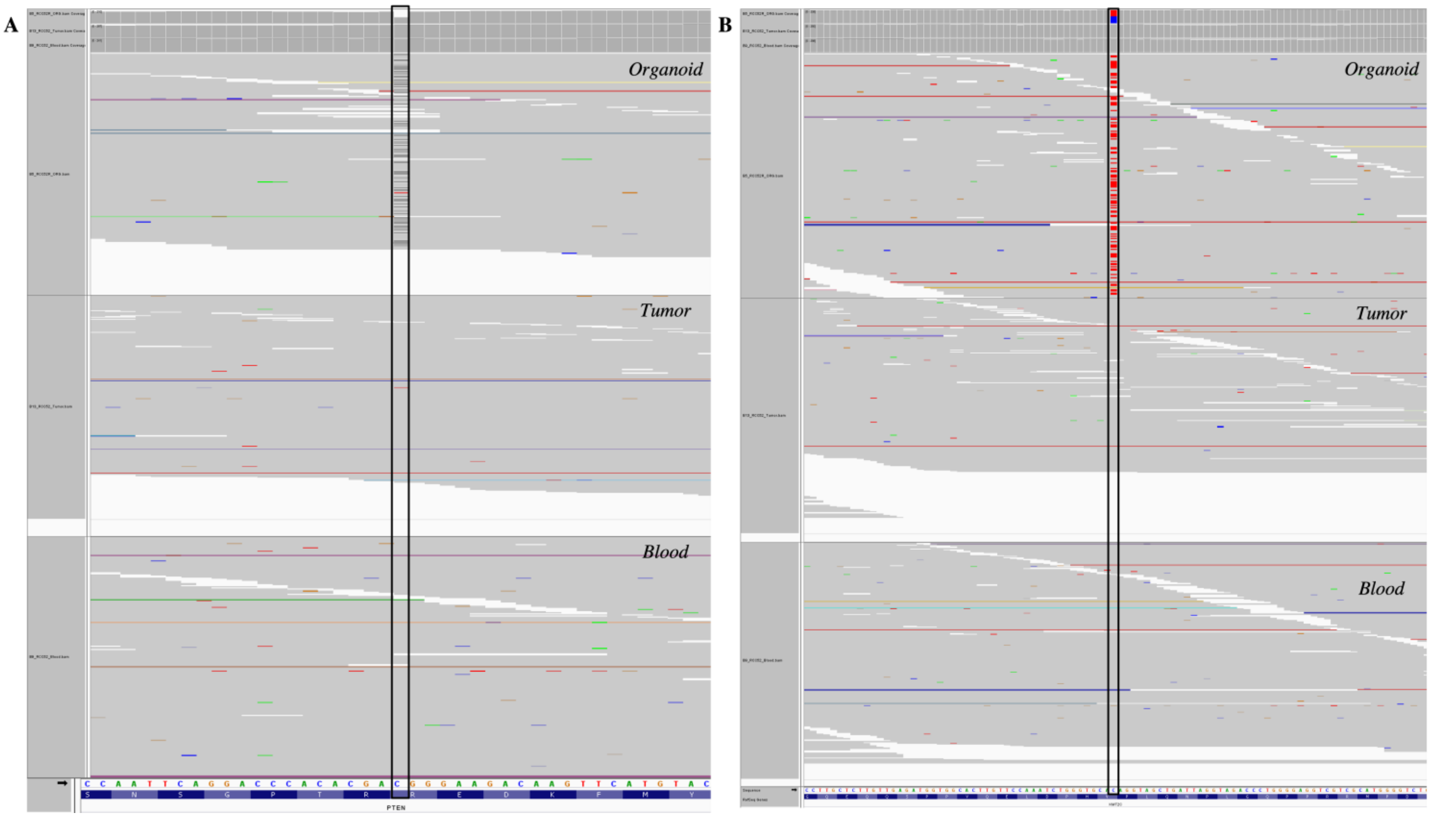

Figure S7. Integrative Genomics Viewer (IGV) genome analysis of RCC52 parental tumor, organoid and blood samples. (A) *PTEN* (CG→C) deletion was found to be specific to organoid only. (B) *KMT2C* mutation (C→T) was found to be specific to organoid only.

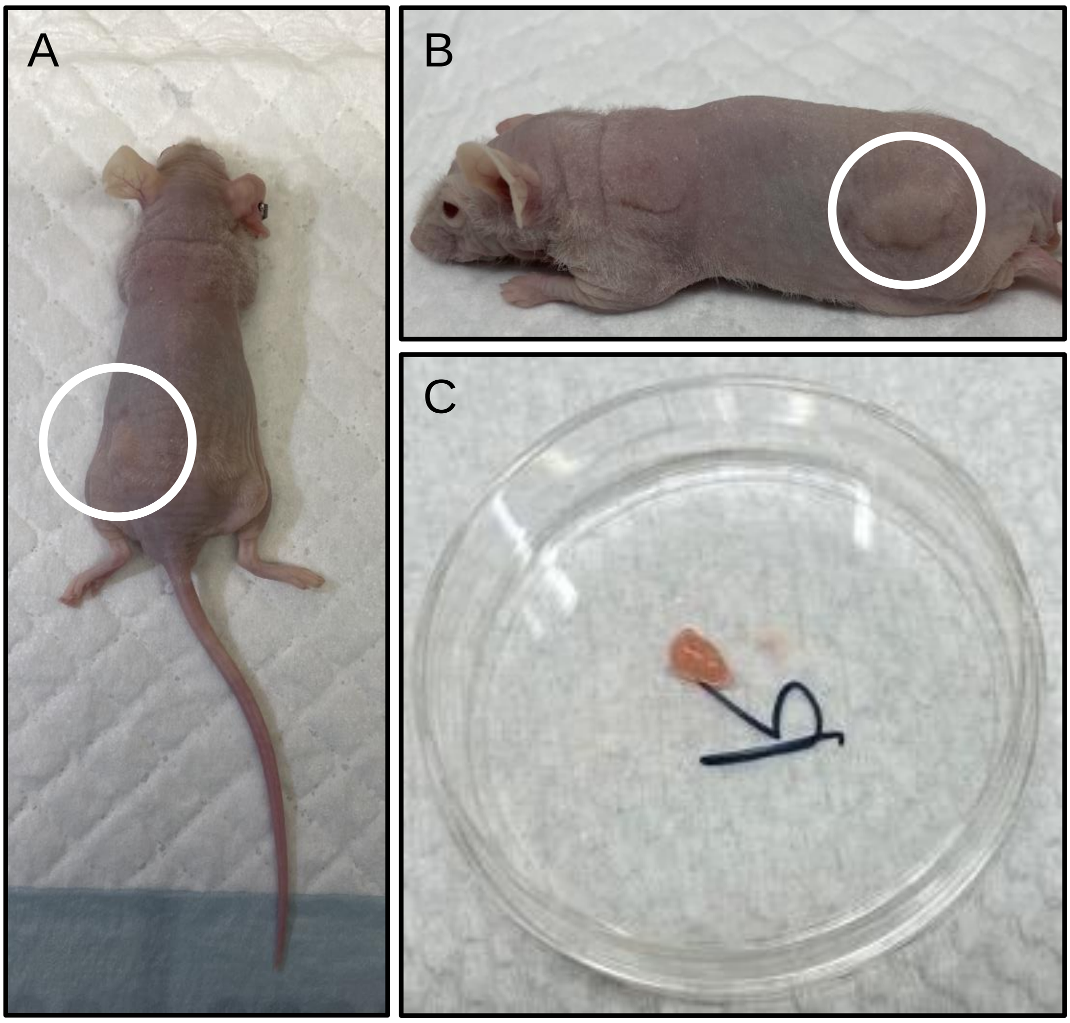

Figure S8. Representative images of subcutaneous growth of tumor in ectopic xenograft model after injection of organoids. (A) Top-view of immunocompromised mice with tumor marked (white circle). (B) Lateral-view of immunocompromised mice with tumor marked (white circle). (C) Tumor harvested from immunocompromised mice.
